# Supplementary material for: Mental Health Outcomes in Australian Healthcare and Aged-Care Workers during the Second Year of the COVID-19 Pandemic
Source: Int J Environ Res Public Health. 2022 Apr 19;19(9):4951. doi: 10.3390/ijerph19094951 (PMC9103405; doi:10.3390/ijerph19094951)
Supplement: Supplementary file 1 [file ijerph-19-04951-s001.zip › ijerph-1667559-supplementary.pdf]

Supplementary Information

Table of Contents

Supplementary Table S1. Perceptions of organisational support in relation to COVID-19 outbreak, as reported by workers of different occupational groups 2

Supplementary Table S2. Demographic characteristics of HCWs providing free text responses+ 3

Supplementary Table S3. Comparison of depression and anxiety data in studies using the same validated instruments as COVIC-HA 4

Supplementary Table S4. Comparison of burnout, PTSD, and resilience data in studies using the same validated instruments as COVIC-HA 5

Supplementary Table S5. Comparison of depression, anxiety, and PTSD data in studies using different instruments 6

Supplementary Table S6. Comparison of alcohol usage data 8

Supplementary Figure S1. COVIC-HA Recruitment Flow Diagram 9

*Supplementary Figure S2. Fear of COVID-19 exposure, workplace concerns, working hours and household income since the start of the pandemic** 10

*Supplementary Figure S3. Social isolation experienced, alcohol consumption levels and mental health support seeking behaviour since the start of the pandemic** 11

Supplementary File S1. Brief introductory survey 12

Supplementary File S2. Baseline Survey 17

Supplementary File S3. Strengthening the reporting of observational studies in epidemiology (STROBE) checklist 44

Supplementary Table S1. Perceptions of organisational support in relation to COVID-19 outbreak, as reported by workers of different occupational groups

Workplace response and supports	Total Cohort* n(%)	Paramedics n(%)	Nurses n(%)	Allied Health n(%)	Doctors n(%)	Others n(%)
			Prevalence of Affirmative responses^			
**Kept me informed on workplace changes**	861 (91.2%)	108 (90.0%)	274 (89.8%)	155 (92.8%)	107 (91.5%)	217 (92.3%)
**Organisation cares about staff welfare**	640 (67.9%)	60 (50.0%)	195 (63.9%)	122 (73.1%)	83 (70.9%)	180 (76.9%)
**Comfortable to voice concerns**	585 (62.0%)	56 (46.7%)	178 (58.4%)	114 (68.3%)	82 (70.1%)	155 (66.2%)
**Organisation responds to concerns**	533 (56.5%)	46 (38.3%)	168 (55.1%)	101 (60.5%)	74 (63.2%)	144 (61.3%)
**Easy access to COVID-19 testing**	767 (81.3%)	40 (33.3%)	272 (89.2%)	148 (88.6%)	104 (89.7%)	203 (86.4%)
**Support services available**	739 (78.3%)	104 (86.7%)	223 (73.1%)	137 (82.0%)	83 (70.9%)	192 (81.7%)
**Access to paid leave for quarantine/isolation**	674 (71.5%)	65 (54.2%)	244 (80.0%)	133 (79.6%)	77 (66.4%)	155 (66.0%)
**Access to accommodation for quarantine/isolation**	505 (53.6%)	54 (45.0%)	195 (64.1%)	83 (49.7%)	64 (54.7%)	109 (46.4%)
**High degree of confidence with current level of PPE**[#]	375 (62.3%)	54 (48.2%)	166 (65.6%)	49 (64.5%)	62 (62.6%)	44 (71.0%)

*Implied totals do not match overall due to non-response

^Data shown represents the number (proportion) of workers who provided affirmative responses, as compared to negative, neutral and "Not applicable" responses

[#]Data shown represents the number (proportion) of workers who indicated they were "Very Confident" or "Confident" (as compared to "Somewhat confident", "A little confident" or "Not confident at all") that the PPE currently available to them is adequate to protect them when managing patients with confirmed or suspected COVID-19

Supplementary Table S2. Demographic characteristics of HCWs providing free text responses+

Characteristics	Free text responses n (%)
**Overall (n)**	**441 (100%)**
**Profession**	
Nurse	153 (35%)
Doctor	54 (12%)
Allied health^	88 (20%)
Paramedic	33 (7%)
Other*	113 (26%)
**Gender**	
Female	343 (78%)
Male	97 (22%)
Non-binary	1 (0.2%)
**Healthcare setting**	
Hospital	383 (87%)
Ambulance	40 (9%)
Primary care	14 (3%)
Aged care	4 (1%)
**Age**	
<45	203 (46%)
≥45	238(54%)

+ Free text responses to question "Please write anything else you would like us to know about your experience of COVID-19"
^ Includes audiology, chiropractic, dietetics, exercise physiology, medial radiation, OT, optometry, orthotics/prosthetics, pharmacy, physiotherapy, psychology, social work, speech pathology
* Includes administrative and clerical staff, orderlies, food service staff, information technology, engineering etc

Supplementary Table S3. Comparison of depression and anxiety data in studies using the same validated instruments as COVIC-HA

Author / Year of publication	Country / State (if Australian)	Study design	Participants	Timing	PHQ-9[α]		GAD-7[α]	
					≥10 (%)	M (SD)	≥10 (%)	M (SD)
**COVIC-HA**	**Australia – Vic[β]**	**Mixed methods Cohort – Baseline findings**	**984 Healthcare and Aged care workers**	**May 2021 – July 2021**	**22.5%**	**6.0 (5.4)**	**14%**	**4.6 (4.7)**
**Australian HCW[δ] studies during COVID-19**								
Dobson et al (2021) [16]	Australia - Vic[β]	Cross-sectional single centre	320 Hospital workers	April – May 2020	21.0%	-	20.0%	-
Roberts et al (2021) [45]	Australia – All states, primarily NSW[γ]	Cross-sectional survey	1,542 frontline workers, including police	-	36.0%	8.2	28.1%	6.8
Smallwood et al (2021) [17]	Australia – All States, primarily Vic[β] (85%)	Cross-sectional online survey	9,518 Frontline healthcare workers	August – October 2020	28.0%	-	28.0%	-
**Australian/New Zealand general population studies during COVID-19**								
Batterham et al (2021) [46]	Australia – All states, highly representative sample	Cohort study, 7 fortnightly surveys	1,296 Australian adults	March – June 2020	18.3% - 23.6%	4.7 (5.6) – 5.8 (6.3)	12.8% - 17.2%	3.7 (4.7) – 4.6 (5.2)
Fisher et al (2020) [26]	Australia – All states, primarily Vic[β] (44%)	Cross-sectional online survey	13, 829 Australian adults	April – May 2020	27.6%	-	21%	-
Fisher et al (2021) [6]	Australia – Vic[β] vs. All other states	Cross-sectional online Survey 1	6,105 Victorian adults	April – May 2020	26.4%	6.8 (95% CI: 6.5; 7)	20.9%	5.5 (95% CI: 5.3; 5.7)
		Cross-sectional online survey 2	4,844 Victorian adults	July – August 2020	44.1%	9.5 (95% CI: 9.2; 9.8)	34.3%	7.7 (95% CI: 7.5; 8)
Gasteiger et al (2021) [47]	New Zealand	Cross-sectional survey	681 New Zealand adults	May – June 2020	31.0%	7.88 (6.4)	24.0%	6.3 (5.4)
**International HCW[δ] studies during COVID-19**								
Choudhoury et al (2020) [48]	United Kingdom	Cross-sectional single centre	106 Hospital healthcare workers	April 2020	15.0%	-	34.0%	-
Hennein et al (2021) [49]	United States	Mixed methods with cross-sectional survey	1,092 Healthcare workers	May 2020	13.9%	-	15.6%	-
Kim et al (2021) [14]	United States	Cross-sectional single centre	320 Registered Nurses	April –May 2020	26.0%	-	43.0%	-
Young et al (2021) [50]	United States	Cross-sectional survey	1,685 Healthcare Workers	April 2020	17.0%	-	33.0%	-

[α]PHQ-9 and GAD-7 scores of <10 indicate minimal-mild symptoms of depression and anxiety respectively, whereas scores of ≥10 indicate moderate-severe symptoms of depression and anxiety (also referred to as clinically significant symptoms)
[β]Vic = Victoria
[γ]NSW = New South Wales
[δ]HCW = Healthcare worker

Supplementary Table S4. Comparison of burnout, PTSD, and resilience data in studies using the same validated instruments as COVIC-HA

Author/year	Country/State (if Australian)	Study Design	Participants	Survey Timing	aMBI			IES-6 ≥9	CD-RISC-2 Mean (SD)
					DP ≥4	EE ≥7	PA ≤14		
**COVIC-HA**	**Australia – Victoria**	**Mixed Methods Cohort – Baseline findings**	**984 Healthcare & aged care workers**	**May – July 2021**	**31%**	**65%**	**45%**	**20.4%**	**6.3 (1.4)**
Smallwood et al (2021) [17]	Australia – All States, primarily Vic (85%)	Cross-sectional online survey	9,518 Frontline healthcare workers	August – October 2020	37%	71%	69%	40.5%	6.4 (1.3) *
Huffman et al (2021) [51]	United States	Cross-sectional survey	785 Healthcare providers	April 2020	-	-	-	-	6.7 (1.2)

*Smallwood et al. presented a mean (SD) CD-RISC-2 score out of 3.21 (0.66) out of 4 (averaged across the two items) rather than out of 8 (as presented in our paper); mean (SD) scores have therefore been adjusted (double) to aid between study comparisons.

Supplementary Table S5. Comparison of depression, anxiety, and PTSD data in studies using different instruments

Author / Year of publication	Country / State (if Australian)	Study design	Participants	Timing	Measurement scale	Depression		Anxiety		PTSD	
						CSS$^{\alpha}$ (%)	M (SD)	CSS$^{\alpha}$ (%)	M (SD)	CSS$^{\alpha}$ (%)	M (SD)
**COVIC-HA**	**Australia – Vic**	**Mixed methods Cohort – Baseline findings**	**984 Healthcare and Aged care workers**	**May 2021 – July 2021**	**PHQ-9, GAD-7, IES-6**	**22.5%**	**6.0 (5.4)**	**14%**	**4.6 (4.7)**	**20.4%**	
**Australian HCW studies during COVID-19**											
Aggar et al (2020) [52]	Australia	Cross-sectional survey	767 Acute care nurses	September – November 2020	DASS-21, IES-R	27.5%	6.71 (9.17)	22%	4.62 (7.37)	17.7%	16.37 (8.04)
Dobson et al (2021) [16]	Australia - Vic	Cross-sectional single centre	320 Hospital workers	April – May 2020	IES-R	-	-	-	-	29%	-
Hammond et al (2021) [53]	International – Primarily Australian (80%)	Cross-sectional online survey	3770 ICU staff	April 2020	DASS-21	21.6%	-	28.6%	-	-	-
Holton et al (2021) [54]	Australia – Vic & Denmark	Cross-sectional online survey	1,611 Australian nurses; 1,390 Danish nurses (not reported here)	May – August 2020	DASS-21	20.8%	-	20.0%	-	-	-
**Australian HCW studies pre-pandemic**											
Axisa et al (2019) [55]	Australia – NSW	RCT – baseline findings	46 Physician trainees	-	DASS-21	52%	-	46%	-	-	-
Beyondblue (2013) [56]	Australia – All states	Cross-sectional survey	11,378 Doctors. 1,811 Med students (not reported here)	-	Self-reported diagnosis of depression or anxiety	20%	-	9%	-	-	-
Creedy et al (2017) [57]	Australia	Cross-sectional survey	1,037 Australian midwives	-	DASS-21	17%	-	20.5%	-	-	-
Hegney et al (2014) [58]	Australia	Cross-sectional single centre	132 Hospital based nurses	May – June 2012	DASS-21	13.6%	2.88 (3.83)	15.2%	2.17 (2.79)		
Maharaj et al (2018) [59]	Australia	Cross-sectional online survey	102 Australian nurses	-	DASS	32.4%	-	41.2%	-	-	-
McGarry (2013) [60]	Australia – WA	Cross-sectional single centre	54 Pediatric health professionals	-	DASS-21, IES-R	10%	2.12 (2.94)	13%	1.48 (2.55)	-	7.43 (7.12)
Pyper & Paterson (2016) [61]	Australia – All states	Mixed Methods – Cross-sectional survey	134 Regional and rural ambulance workers	-	IES	-	-	-	-	15.4%	-
**Australian/New Zealand general population studies during COVID-19**											
Newby et al (2020) [36]	Australia – All states, representative sample	Cross-sectional online survey	5,071 Australian adults	March – April 2020	DASS-21	46.3%	-	40.8%	-	-	-
Rossell et al (2020) [62]	Australia – All states, primarily Vic (61.8%)	Cross-sectional online survey	5,545 Australian adults	April 2020	DASS-21	29.0%	-	23.0%	-	-	-
Stanton et al (2020) [63]	Australia	Cross-sectional online survey	1,491 Australian adults	April 2020	DASS-21	26.6%	-	13.5%	-	-	-
**International HCW studies during COVID-19**											
Marvaldi et al (2021) [11]	International – Primarily China	Systematic review & meta-analysis	101,017 Healthcare workers	Existing literature to Oct2020	Multiple VI's – Random effects model	31.1%	-	30%	-	20.2%	-
Sanghera et al (2020) [64]	International – Primarily China	Systematic review	69,499 Hospital based workers	Dec 2019 to June 2020	Multiple VI's – Random effects model	13.5% – 44.7%	-	12.3% – 35.6%	-	7.4% – 37.4%	-
Saragih et al (2021) [10]	International – Primarily China	Systematic review & meta-analysis	53,784 Healthcare workers	Dec 2019 to Nov 2020	Multiple VI's – Random effects model	37%	-	40%	-	49%	-

Serrano-Ripoll et al (2020) [65]	International – Primarily Asia	Rapid systematic review & meta-analysis	119,189 Healthcare workers	Existing literature to August 2020	Multiple VI's – Random effects model	24%	-	30%	-	13%	-

[α]CSS=Clinically significant symptoms, defined as moderate-severe/extremely severe on the above validated instruments

Supplementary Table S6. Comparison of alcohol usage data

Author/year	Country/State (if Australian)	Study Design	Participants	Survey Timing	Change in alcohol use since the beginning of the pandemic		
					Increased (%)	Stayed the same (%)	Decreased (%)
**COVIC-HA**	**Australia – Victoria**	**Mixed Methods Cohort – Baseline findings**	**984 Healthcare & aged care workers**	**May – July 2021**	**27.2%**	**43.7%**	**14.9%**
Tran et al (2020) [66]	Australia – All states, primarily Vic, (44%)	Cross-sectional online survey	13,829 Australian adults	April – May 2020	20.9%	43.9%	10.5%
Rahman et al (2020) [α] [67]	Australia – All states, primarily Vic (88.2%)	Cross-sectional online survey	587 Australian adults, primarily FW[γ] workers (42.3%)	June 2020	31.4%	68.6%	-
Bhoyroo et al (2021) [β] [68]	Australia – WA[δ]	Cross-sectional online survey	547 Western Australian adults	August – October 2020	35.1%	66.4%	10.5%
Stanton et al (2020) [63]	Australia – All states	Cross-sectional online survey	1,491 Australian adults	April 2020	26.6%	55.3%	18.1%

[α]Rahman et al assessed change in alcohol use over the last 4 weeks

[β]Bhoyroo et al assessed change in alcohol use pre and post 3-month Western Australian lockdown

[γ]FW=Frontline workers

[δ]WA=Western Australia

Supplementary Figure S1. COVIC-HA Recruitment Flow Diagram

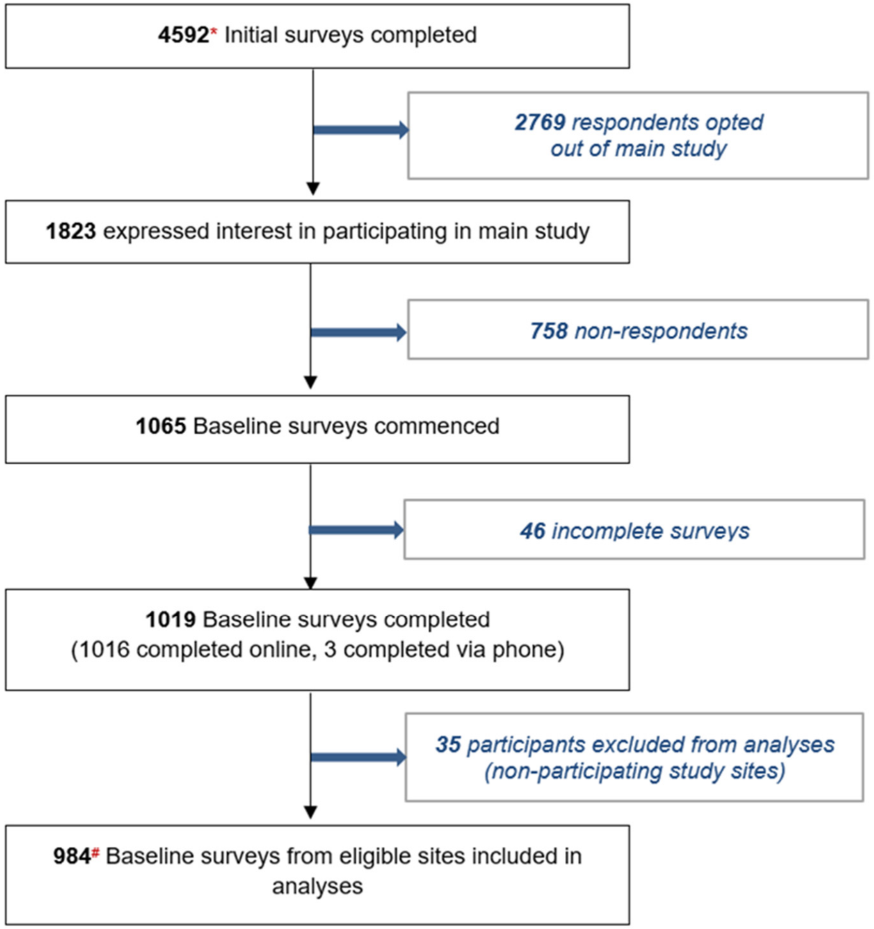

*Of the 4592, 75.7% were female, 53.1% aged <45 years and 80.6% were neither infected nor furloughed

#Of the 984, 72.6% were female, 50.7% aged <45 years and 77.1% were neither infected nor furloughed

Supplementary Figure S2. Fear of COVID-19 exposure, workplace concerns, working hours and household income since the start of the pandemic*

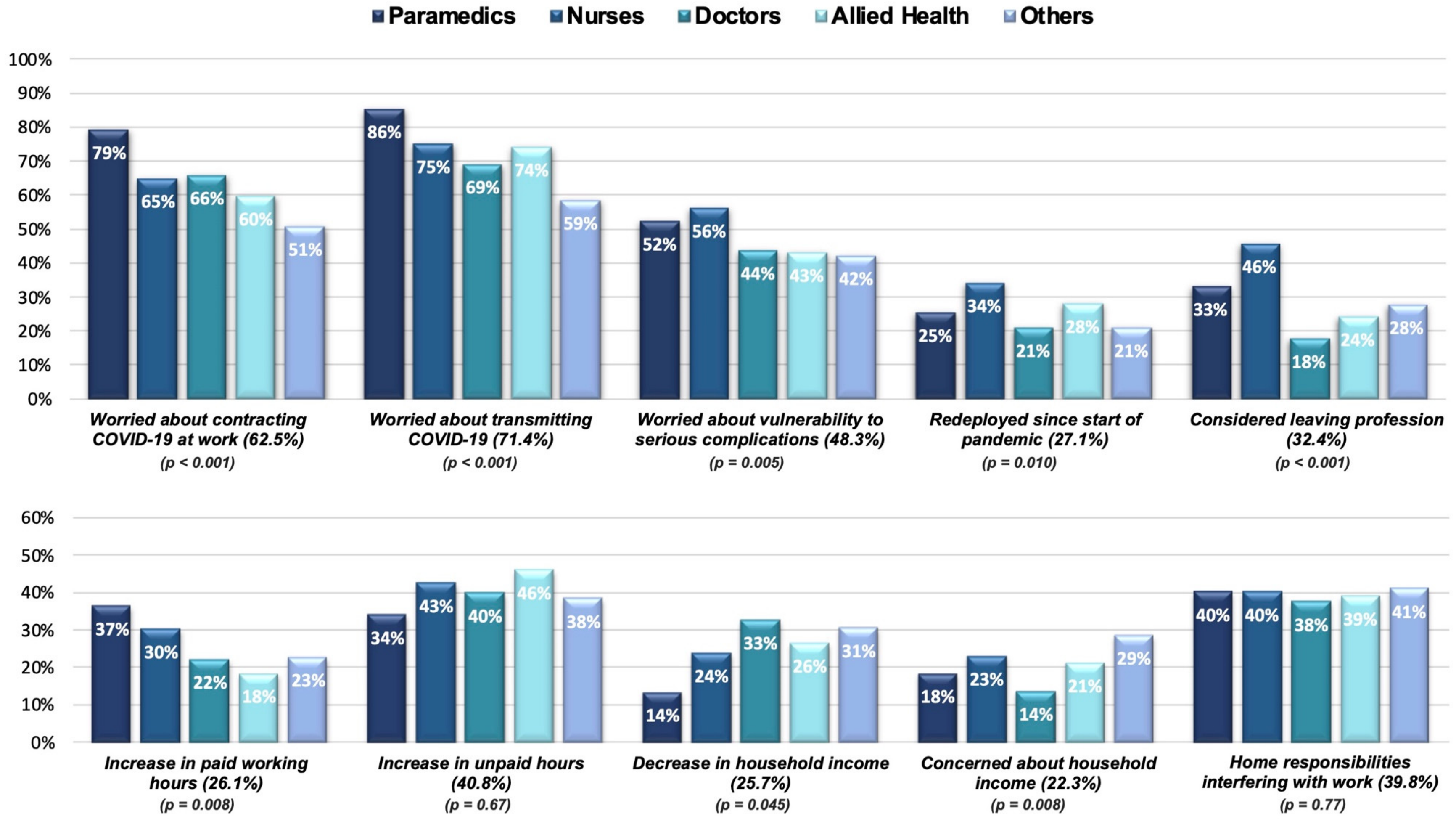

*Relative risks can be found in Table 3 in the main manuscript. P-values correspond to the test of the global null hypothesis of no difference between occupations

Percentages in brackets represent proportion of total cohort answering "yes" to each question

Supplementary Figure S3. Social isolation experienced, alcohol consumption levels and mental health support seeking behaviour since the start of the pandemic*

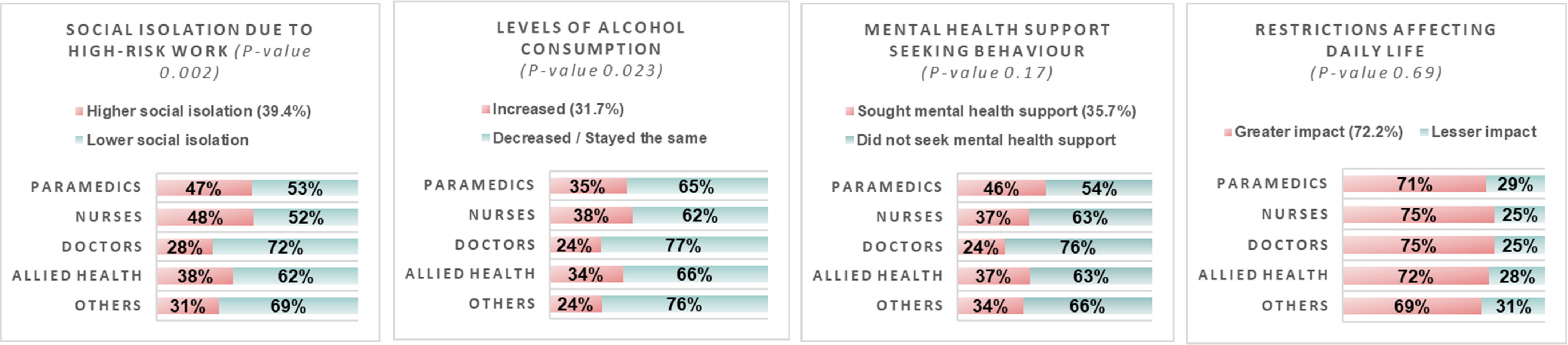

*Relative risks can be found in Table 3 in the main manuscript. P-values correspond to the test of the global null hypothesis of no difference between occupations

Supplementary File S1. Brief introductory survey

Resize font:

Initial Survey

Please complete the survey below.

Thank you!

Part A: About you and your work

This group of questions asks about you and your work situation

How old are you (in years)?

* must provide value

What is your gender?

* must provide value

- Female
- Male
- Non-binary/gender diverse/other
- Prefer not to say

reset

What is your profession/work background?

* must provide value

Please select the MAIN setting in which you currently work
* must provide value

- ◯ Hospital (inpatient and/or outpatient)
- ◯ Primary care (e.g. general practice)
- ◯ Aged care (e.g. residential aged care facility)
- ◯ Ambulance service
- ◯ Other

reset

Please select the MAIN organisation that you work for
* must provide value

Part B: Experience of COVID-19

This next set of questions asks about your experience of COVID-19.

Have you ever been tested for COVID-19?
* must provide value

◯ Yes ◯ No

reset

Have you ever been quarantined (furloughed) due to potential exposure to someone with COVID-19?

- ◯ Yes - exposed at work
- ◯ Yes - exposed outside of work
- ◯ No

reset

Part C: Your opinions and concerns

This final set of questions asks about your opinions and concerns regarding COVID-19. Please answer these questions for the healthcare organisation where you mainly work.

Please indicate the extent to which you agree with the following statements:

	Strongly disagree	Somewhat disagree	Neither agree nor disagree	Somewhat agree	Strongly agree	Not Applicable
**I am worried about being exposed to COVID-19 at work** * must provide value	○	○	○	○	○	○ reset
**I am worried that I may transmit COVID-19 to others, such as friends and family, because of my work** * must provide value	○	○	○	○	○	○ reset
**I am worried that I would be vulnerable to serious complications if I contracted COVID-19** * must provide value	○	○	○	○	○	○ reset

Part D: Interest in participating in the COVIC-HA Study

We now ask that you consider whether you would be interested in participating in the COVIC-HA study, which is described in the following explanatory statement. Please indicate your interest in participating in the study by selecting the appropriate response(s) on the form provided at the end of the explanatory statement.

EXPLANATORY STATEMENT

Healthcare and aged care workers - main study

Project ID: 68086

Project: Coronavirus in Victorian Healthcare and Aged care workers (COVIC-HA) Cohort Study

You are invited to take part in the COVIC-HA cohort study which aims to examine the ongoing effects of the COVID-19 pandemic on the health and wellbeing of people who work in the healthcare and aged care settings in Victoria, and the preparedness and responses of their workplaces.

This study involves four elements.

1. Completion of two surveys (questionnaires), six months apart
2. Linkage of survey data to various health datasets (data linkage)
3. Blood testing to look for evidence of antibodies to the COVID-19 virus
4. Participation in an interview to explore your personal experiences of COVID-19

All people who agree to take part in this study will be invited to participate in elements 1 and 2, and some people taking part in this study will be invited to participate in elements 3 and 4. Please read the **Explanatory Statement** in full before deciding whether or not to participate.

If you have any questions or would like any further information about any aspect of this study, please contact the study team at covicha@monash.edu, or visit our website www.monash.edu/covic-ha.

Please indicate your interest in participating in the survey and/or data linkage elements of the COVIC-HA Cohort Study by selecting ONE of the following options:

* must provide value

- ○ I am interested in participating in the COVIC-HA study and provide consent for the survey responses and my contact details to be shared with study investigators.
- ○ I am not interested in participating in the COVIC-HA study and wish for the survey responses I have provided to remain anonymous

reset

Submit

Part A: About you

This first group of questions asks about you and your home situation.

What is your residential postcode?

* must provide value

Date of birth:

* must provide value

D-M-Y

DD/MM/YYYY

What is your gender?

* must provide value

- ○ Female
- ○ Male
- ○ Non-binary / gender diverse / other
- ○ Prefer not to say

reset

What is your country of birth?

* must provide value

Is English your first language?

* must provide value

- ◯ Yes
- ◯ No

reset

Are you :

* must provide value

- ◯ An Australian Citizen
- ◯ A permanent resident
- ◯ A short term visa holder
- ◯ Prefer not to say

reset

Are you of Aboriginal and Torres Strait Islander origin? (select one option)

* must provide value

- ◯ Aboriginal
- ◯ Torres Strait Islander
- ◯ Both Aboriginal and Torres Strait Islander
- ◯ Neither
- ◯ Prefer not to say

reset

Do you live: (select all that apply)

* must provide value

- ☐ On your own
- ☐ With your partner/spouse
- ☐ With children (< 18yrs)
- ☐ With adult family members (eg. mother, father, brother, sister, children >18yrs)
- ☐ With adult non-family members (eg. housemates, friends)
- ☐ Other, please specify

At any stage of the pandemic, have changes in home schooling or caring responsibilities related to COVID-19 interfered with your ability to work compared to pre-pandemic levels?

* must provide value

- ○ Yes, major impact
- ○ Yes, some impact
- ○ No
- ○ Not applicable

reset

Since the COVID-19 pandemic started, how much have the COVID-19 restrictions affected your daily life?

* must provide value

No effect 0 — 5 — Very large effect 10

Change the slider above to set a response

reset

Survey Completeness

10%

Part B: Your work situation

This next group of questions asks about your work situation and background.

At any stage since the start of the COVID-19 pandemic, have you worked in a health care setting (including hospital, aged care, primary care, ambulance)?

* must provide value

- ◯ Yes
- ◯ No

reset

In the past TWO weeks, have you worked in a health care setting (including hospital, aged care, primary care, ambulance)?

* must provide value

- ◯ Yes
- ◯ No

reset

Please select the MAIN setting in which you currently work

* must provide value

- ◯ Hospital (inpatient and/or outpatient)
- ◯ Primary care (e.g. general practice)
- ◯ Aged care (e.g. residential aged care facility)
- ◯ Ambulance service
- ◯ None of the above

reset

What is your MAIN profession or work background?

* must provide value

- ◯ Doctor
- ◯ Nurse
- ◯ Allied Health
- ◯ Paramedic
- ◯ Personal care assistant
- ◯ Clinical scientist/technician
- ◯ Clerical or administrative
- ◯ Food services
- ◯ Cleaning services
- ◯ Engineering services
- ◯ Research
- ◯ Student - medical, nursing, allied health
- ◯ Corporate/management
- ◯ Other, please specify

reset

How many years have you worked in your current profession (i.e. since graduating from university or completing your certification):

* must provide value

- ◯ 0-5 years
- ◯ 6-10 years
- ◯ 11-15 years
- ◯ More than 15 years

reset

AS BEST AS POSSIBLE, PLEASE ANSWER THE FOLLOWING SECTIONS BASED ON YOUR EXPERIENCE AT THE MAIN ORGANISATION THAT YOU WORK FOR.

Before the COVID-19 pandemic what was your total employment status?

* must provide value

- ○ Full time
- ○ Part time
- ○ Casual
- ○ Other

reset

What is your CURRENT employment status (taking into consideration all jobs you have)?

* must provide value

- ○ Full time
- ○ Part time
- ○ Casual
- ○ Other

reset

At any stage since the start of the COVID-19 pandemic, did your paid working hours:

* must provide value

- ○ Increase
- ○ Decrease
- ○ Stay the same

reset

At any stage since the start of the COVID-19 pandemic, on average, did the hours you spent doing unpaid work (e.g. home-schooling, providing care, household tasks):
* must provide value

- ◯ Increase
- ◯ Decrease
- ◯ Stay the same

reset

Since the start of the COVID-19 pandemic has your average household income altered?
* must provide value

- ◯ Increased
- ◯ Decreased
- ◯ Stayed the same

reset

As a result of COVID-19 do you have concerns or worries about your household income?
* must provide value

- ◯ Yes
- ◯ No

reset

As a result of COVID-19, have you considered leaving your role/profession?
* must provide value

- ◯ Yes
- ◯ No

reset

At any time since the start of the COVID-19 pandemic, have you been deployed (sent into) to a new area of work?
* must provide value

- ◯ Yes
- ◯ No

reset

25% complete

Part C: Your general health

This next group of questions asks about your general health

In general, would you say your current health is...

* must provide value

○ Excellent ○ Very good ○ Good ○ Fair ○ Poor

reset

Prior to the COVID-19 pandemic, would you say your health was...

* must provide value

○ Excellent ○ Very good ○ Good ○ Fair ○ Poor

reset

Do you know your BMI?

BMI = body mass index.

* must provide value

○ Yes

○ No

reset

**Estimated weight (kg)**	
**Estimated height (cm)**	
**Calculated BMI**	

Do you have any of the following health conditions? (please select all that apply)

* must provide value

- ☐ Diabetes Type I
- ☐ Diabetes Type II
- ☐ Cancer
- ☐ Heart disease (e.g. coronary artery disease or heart failure)
- ☐ High blood pressure (hypertension)
- ☐ Chronic lung disease (excluding mild or moderate asthma)
- ☐ Chronic kidney disease
- ☐ Chronic liver disease
- ☐ Organ or bone marrow transplant
- ☐ A condition for which you take regular immunosuppressive medications (e.g. steroids)
- ☐ Pregnancy
- ☐ None of the above

Do you smoke?

* must provide value

- ○ Yes
- ○ No

Compared to before the COVID-19 pandemic, on average have you been drinking alcohol:

* must provide value

- ◯ More than you used to
- ◯ Less than you used to
- ◯ About the same
- ◯ Not applicable (I don't drink alcohol)

reset

Before the COVID-19 pandemic, had you ever sought help for stress, anxiety, depression or another mental health issue?

* must provide value

- ◯ Yes
- ◯ No
- ◯ Prefer not to say

reset

Since the COVID-19 pandemic started, have you sought help for stress, anxiety, depression or another mental health issue?

* must provide value

- ◯ Yes
- ◯ No
- ◯ Prefer not to say

reset

Since the COVID-19 pandemic started, have you done any of the following to help with stress, anxiety or low mood? (please select all that apply)

* must provide value

- ☐ Maintained or increased exercise
- ☐ Practiced yoga, meditation, mindfulness or gratitude
- ☐ Used voice calls, video calls, or messaging to stay connected with friends and/or family
- ☐ Spent additional time on hobbies, or took up a new hobby
- ☐ Avoided or took breaks from the news
- ☐ Started a new prescription medication
- ☐ Taken up or increased smoking
- ☐ Used recreational drugs
- ☐ Increased alcohol intake
- ☐ Other strategy
- ☐ Not applicable

40% complete

Part D: Care of patients with COVID-19

This next group of questions asks about your interactions with patients with confirmed or suspected COVID-19 and your confidence and training in the use of personal protective equipment (PPE)

In your work, have you EVER interacted (in person) with patients with confirmed or suspected COVID-19 infection?

* must provide value

- ○ Yes, confirmed COVID-19 cases only
- ○ Yes, suspected COVID-19 cases only
- ○ Yes, both confirmed and suspected COVID-19 cases
- ○ Not that I know of

reset

Have you received formal training in the use of Personal Protective Equipment (PPE) at your institution?

(select all that apply)

* must provide value

- ☐ Yes, at commencement of employment at my current institution
- ☐ Yes, since the start of the COVID-19 pandemic
- ☐ No
- ☐ Not applicable

Do you believe that you would benefit from further Personal Protective Equipment (PPE) training and education?

(select all that apply)

* must provide value

- ☐ Yes- lectures and printed materials
- ☐ Yes- demonstration by infection control experts
- ☐ Yes- simulation-based training
- ☐ Yes - online modules
- ☐ No
- ☐ Not applicable

50% complete

Part E: Experience of COVID-19

This next group of questions asks about your personal experiences of COVID-19.

NOTE if using a phone to complete this survey, some questions will be more mobile-friendly if phone is turned landscape

Have you ever been tested for COVID-19?

* must provide value

- ○ Yes
- ○ No

reset

Has anyone in your household been diagnosed with COVID-19? (select all that apply)

* must provide value

- ☐ Yes, family member
- ☐ Yes, non-family, household member
- ☐ No
- ☐ I don't know

60% complete

At any point since the start of the pandemic, please indicate the extent to which you agree with the following statements:

	Strongly disagree	Somewhat disagree	Neither agree nor disagree	Somewhat agree	Strongly agree	Not applicable
**I have been worried about being exposed to COVID-19 at work** * must provide value	◯	◯	◯	◯	◯	◯ reset
**I have been worried that I may transmit COVID-19 to others, such as friends and family, because of my work** * must provide value	◯	◯	◯	◯	◯	◯ reset
**I have been worried that I would be vulnerable to serious complications if I contracted COVID-19** * must provide value	◯	◯	◯	◯	◯	◯ reset
**I feel that my family or friends have avoided contact with me because I work in a 'high-risk' environment** * must provide value	◯	◯	◯	◯	◯	◯ reset

63% complete

Please indicate the extent to which you agree with the following statements CURRENTLY:

	Strongly disagree	Somewhat disagree	Neither agree nor disagree	Somewhat agree	Strongly agree	Not applicable
**I am worried about being exposed to COVID-19 at work** * must provide value	◯	◯	◯	◯	◯	◯ reset
**I am worried that I may transmit COVID-19 to others, such as friends and family, because of my work** * must provide value	◯	◯	◯	◯	◯	◯ reset
**I am worried that I would be vulnerable to serious complications if I contracted COVID-19** * must provide value	◯	◯	◯	◯	◯	◯ reset
**I feel that my family or friends avoid contact with me because I work in a 'high-risk' environment** * must provide value	◯	◯	◯	◯	◯	◯ reset

Did you receive the seasonal influenza vaccine in 2020?

* must provide value

◯ Yes

◯ No

reset

Have you received one or more doses of a coronavirus (COVID-19) vaccine?

* must provide value

○ Yes

○ No

reset

68% complete

Please indicate the extent to which you agree with the following statements:

	Strongly disagree	Somewhat disagree	Neither agree not disagree	Somewhat agree	Strongly agree
**All healthcare workers should be vaccinated against COVID-19** * must provide value	○	○	○	○	○
**It is important that healthcare workers have freedom of choice in vaccination** * must provide value	○	○	○	○	○

reset

reset

70% complete

Part F: Stress, anxiety and professional fulfilment
This section asks a series of questions about your feelings, thoughts and experiences using well validated scales.

Over the last TWO weeks, how often have you been bothered by any of the following problems?

	Not at all	Several days	More than half the days	Nearly every day
**Little interest or pleasure in doing things**<br>* must provide value	○	○	○	○ reset
**Feeling down, depressed, or hopeless**<br>* must provide value	○	○	○	○ reset
**Trouble falling or staying asleep, or sleeping too much**<br>* must provide value	○	○	○	○ reset
**Feeling tired or having little energy**<br>* must provide value	○	○	○	○ reset
**Poor appetite or overeating**<br>* must provide value	○	○	○	○ reset
**Feeling bad about yourself, or that you are a failure, or have let yourself or your family down**<br>* must provide value	○	○	○	○ reset

Trouble concentrating on things, such as reading the newspaper or watching television
* must provide value

reset

Moving or speaking so slowly that other people could have noticed. Or the opposite, being so fidgety or restless that you have been moving around a lot more than usual
* must provide value

reset

Thoughts that you would be better off dead, or of hurting yourself in some way
* must provide value

reset

75% complete

Over the last TWO weeks, how often have you been bothered by any of the following problems?

	Not at all	Several days	More than half the days	Nearly every day
**Feeling nervous, anxious, or on edge** * must provide value	◯	◯	◯	◯ reset
**Not being able to stop or control worrying** * must provide value	◯	◯	◯	◯ reset
**Worrying too much about different things** * must provide value	◯	◯	◯	◯ reset
**Trouble relaxing** * must provide value	◯	◯	◯	◯ reset
**Being so restless that it is hard to sit still** * must provide value	◯	◯	◯	◯ reset
**Becoming easily annoyed or irritable** * must provide value	◯	◯	◯	◯ reset
**Feeling afraid as if something awful might happen** * must provide value	◯	◯	◯	◯ reset

80% complete

Over the last TWO weeks, how has the COVID-19 pandemic impacted your everyday thinking?

	Not at all	A little bit	Moderately	Quite a bit	Extremely
**I thought about it when I didn't mean to** * must provide value	◯	◯	◯	◯	◯ reset
**Other things kept making me think about it** * must provide value	◯	◯	◯	◯	◯ reset
**I was aware that I still had a lot of feelings about it, but I didn't deal with them** * must provide value	◯	◯	◯	◯	◯ reset
**I tried not to think about it** * must provide value	◯	◯	◯	◯	◯ reset
**I felt watchful or on guard** * must provide value	◯	◯	◯	◯	◯ reset
**I had trouble concentrating** * must provide value	◯	◯	◯	◯	◯ reset

For each of the following statements, mark the box that most accurately reflects your response

	Every day	A few times a week	Once a week	A few times a month	Once a month or less	A few times a year	Never	Not applicable
**I deal very effectively with the problems of my patients/residents** * must provide value	○	○	○	○	○	○	○	○ reset
**I feel I treat some patients/residents as if they were impersonal objects** * must provide value	○	○	○	○	○	○	○	○ reset
**I feel emotionally drained from my work** * must provide value	○	○	○	○	○	○	○	○ reset
**I feel fatigued when I get up in the morning and have to face another day on the job** * must provide value	○	○	○	○	○	○	○	○ reset
**I've become more callous towards people since I took this job** * must provide value	○	○	○	○	○	○	○	○ reset

reset

I feel I'm positively influencing other people's lives through my work
* must provide value

reset

Working with people all day is really a strain for me
* must provide value

reset

I don't really care what happens to some patients
* must provide value

reset

I feel exhilarated after working closely with my patients/residents
* must provide value

reset

85% complete

Part G: Wellbeing and resilience

This section asks a series of questions about how satisfied you currently feel, and how well-equipped you feel to bounce back or recover from stress, using well-validated scales.

The following questions are you how satisfied you feel on a scale from 0 to 10.

Zero means you feel no satisfaction at all and 10 means you are completely satisfied.

	0 (Not satisfied at all)	1	2	3	4	5	6	7	8	9	10 (Completely satisfied)
**Thinking about your own life and personal circumstances, how satisfied are you with your life as a whole?** * must provide value	○	○	○	○	○	○	○	○	○	○	○ reset
**How satisfied are you with your standard of living?** * must provide value	○	○	○	○	○	○	○	○	○	○	○ reset
**How satisfied are you with your health?** * must provide value	○	○	○	○	○	○	○	○	○	○	○ reset

How satisfied are you with what you are achieving in life?
* must provide value

reset

How satisfied are you with your personal relationships?
* must provide value

reset

How satisfied are you with how safe you feel?
* must provide value

reset

How satisfied are you with feeling part of your community?
* must provide value

reset

How satisfied are you with your future security?
* must provide value

reset

90% complete

Please indicate the extent to which the following statements apply to you.

	Not true at all	Rarely true	Sometimes true	Often true	True nearly all the time
**I am able to adapt when changes occur** * must provide value	◯	◯	◯	◯	◯ reset
**I tend to bounce back after illness, injury, or other hardships** * must provide value	◯	◯	◯	◯	◯ reset

Please tell us in general how optimistic you feel about the future.

Zero means ***Not at all optimistic*** *and 10 means* ***Extremely optimistic***

* must provide value

0 5 10

Change the slider above to set a response

reset

Please write anything else you would like us to know about your experience of COVID-19 (up to 250 characters)

Part H: Workplace response and supports

This final group of questions ask about your opinions about your healthcare organisation's response to the COVID-19 pandemic and the resources and supports made available to you. Please answer these questions for the healthcare organisation where you mainly work.

Please indicate the extent to which you agree with the following statements:

	Strongly disagree	Somewhat disagree	Neither agree nor disagree	Somewhat agree	Strongly agree	Not applicable
**My organisation has kept me informed about workplace changes in response to COVID-19** * must provide value	○	○	○	○	○	○ reset
**My organisation cares about my welfare** * must provide value	○	○	○	○	○	○ reset
**I feel comfortable to voice my concerns within my organisation** * must provide value	○	○	○	○	○	○ reset
**My organisation responds to concerns raised by workers** * must provide value	○	○	○	○	○	○ reset
**My organisation provides easy access to COVID-19 testing for staff** * must provide value	○	○	○	○	○	○ reset

There are services in place at my organisation to support my wellbeing and mental health (e.g. telephone support lines, wellness checks, apps)
* must provide value

reset

If I need to isolate or quarantine due to COVID-19, my organisation will provide me with paid leave
* must provide value

reset

If I need to isolate or quarantine away from my family due to COVID-19, my organisation will help me access accommodation (e.g. via the Hotels for Heroes program)
* must provide value

reset

What resources or supports would you like to see your organisation put into place to support you during the COVID-19 pandemic and any future crisis events? (up to 250 characters, use of dot points welcome)

Expand

Supplementary File S3. Strengthening the reporting of observational studies in epidemiology (STROBE) checklist

	Item No	Recommendation	Page No	Relevant section from manuscript
**Title and abstract**	1	(*a*) Indicate the study's design with a commonly used term in the title or the abstract	1	Abstract
		(*b*) Provide in the abstract an informative and balanced summary of what was done and what was found	1	Abstract
**Introduction**				
Background/rationale	2	Explain the scientific background and rationale for the investigation being reported	1-2	Introduction
Objectives	3	State specific objectives, including any prespecified hypotheses	2-3	Introduction
**Methods**				
Study design	4	Present key elements of study design early in the paper	3	Methods
Setting	5	Describe the setting, locations, and relevant dates, including periods of recruitment, exposure, follow-up, and data collection	3	Methods
Participants	6	(*a*) Give the eligibility criteria, and the sources and methods of selection of participants. Describe methods of follow-up	3	Methods
		(*b*) For matched studies, give matching criteria and number of exposed and unexposed		
Variables	7	Clearly define all outcomes, exposures, predictors, potential confounders, and effect modifiers. Give diagnostic criteria, if applicable	3-4	Methods

Data sources/ measurement	8*	For each variable of interest, give sources of data and details of methods of assessment (measurement). Describe comparability of assessment methods if there is more than one group	3-4	Methods
Bias	9	Describe any efforts to address potential sources of bias	3-4,8	Methods, Discussion
Study size	10	Explain how the study size was arrived at	3,8, Figure S1	Methods, Discussion, Supplementary Appendix
Quantitative variables	11	Explain how quantitative variables were handled in the analyses. If applicable, describe which groupings were chosen and why	3-4	Methods
Statistical methods	12	(*a*) Describe all statistical methods, including those used to control for confounding<br>(*b*) Describe any methods used to examine subgroups and interactions<br>(*c*) Explain how missing data were addressed<br>(*d*) If applicable, explain how loss to follow-up was addressed<br>(*e*) Describe any sensitivity analyses	3-4	Methods
**Results**				
Participants	13*	(a) Report numbers of individuals at each stage of study—eg numbers potentially eligible, examined for eligibility, confirmed eligible, included in the study, completing follow-up, and analysed<br>(b) Give reasons for non-participation at each stage<br>(c) Consider use of a flow diagram	4, Figure S1	Results, Supplementary Appendix

Descriptive data	14*	(a) Give characteristics of study participants (eg demographic, clinical, social) and information on exposures and potential confounders<br><br>(b) Indicate number of participants with missing data for each variable of interest<br><br>(c) Summarise follow-up time (eg, average and total amount)	4, Table 1	Results, Tables
Outcome data	15*	Report numbers of outcome events or summary measures over time	4-8	Results
Main results	16	(*a*) Give unadjusted estimates and, if applicable, confounder-adjusted estimates and their precision (eg, 95% confidence interval). Make clear which confounders were adjusted for and why they were included<br><br>(*b*) Report category boundaries when continuous variables were categorized	4-8, Tables 2-3	Results, Tables
		(*c*) If relevant, consider translating estimates of relative risk into absolute risk for a meaningful time period		
Other analyses	17	Report other analyses done—eg analyses of subgroups and interactions, and sensitivity analyses	n/a	n/a
**Discussion**				
Key results	18	Summarise key results with reference to study objectives	9	Discussion
Limitations	19	Discuss limitations of the study, taking into account sources of potential bias or imprecision. Discuss both direction and magnitude of any potential bias	10-11	Discussion
Interpretation	20	Give a cautious overall interpretation of results considering objectives, limitations, multiplicity of analyses, results from similar studies, and other relevant evidence	9-11	Discussion
Generalisability	21	Discuss the generalisability (external validity) of the study results	10-11	Discussion

**Other information**				
Funding	22	Give the source of funding and the role of the funders for the present study and, if applicable, for the original study on which the present article is based	12	

*Give information separately for exposed and unexposed groups.

Note: An Explanation and Elaboration article discusses each checklist item and gives methodological background and published examples of transparent reporting. The STROBE checklist is best used in conjunction with this article (freely available on the Web sites of PLoS Medicine at http://www.plosmedicine.org/, Annals of Internal Medicine at http://www.annals.org/, and Epidemiology at http://www.epidem.com/). Information on the STROBE Initiative is available at http://www.strobe-statement.org.

Referneces

1. Roberts, N.J.; McAloney-Kocaman, K.; Lippiett, K.; Ray, E.; Welch, L.; Kelly, C. Levels of resilience, anxiety and depression in nurses working in respiratory clinical areas during the COVID pandemic. *Respir. Med.* **2020**, *176*, 106219.
2. Batterham, P.J.; Calear, A.L.; McCallum, S.M.; Morse, A.R.; Banfield, M.; Farrer, L.M.; Gulliver, A.; Cherbuin, N.; Harris, R.M.R.; Shou, Y.; et al. Trajectories of depression and anxiety symptoms during the COVID-19 pandemic in a representative Australian adult cohort. *Med. J. Aust.* **2021**, *214*, 462–468.
3. Gasteiger, N.; Vedhara, K.; Massey, A.; Jia, R.; Ayling, K.; Chalder, T.; Coupland, C.; Broadbent, E. Depression, anxiety and stress during the COVID-19 pandemic: Results from a New Zealand cohort study on mental well-being. *BMJ Open* **2021**, *11*, e045325.
4. Choudhury, T.; Debski, M.; Wiper, A.; Abdelrahman, A.; Wild, S.; Chalil, S.; More, R.; Goode, G.; Patel, B.; Abdelaziz, H.K. COVID-19 Pandemic: Looking After the Mental Health of Our Healthcare Workers. *J. Occup. Environ. Med.* **2020**, *62*, e373–e376.
5. Hennein, R.; Mew, E.J.; Lowe, S.R. Socio-ecological predictors of mental health outcomes among healthcare workers during the COVID-19 pandemic in the United States. *PLoS ONE* **2021**, *16*, e0246602.
6. Young, K.P.; Kolcz, D.L.; O'Sullivan, D.M.; Ferrand, J.; Fried, J.; Robinson, K.C. Health Care Workers' Mental Health and Quality of Life During COVID-19: Results from a Mid-Pandemic, National Survey. *Psychiatr. Serv.* **2021**, *72*, 122–128.
7. Huffman, E.M.; Athanasiadis, D.I.; Anton, N.E.; Haskett, L.A.; Doster, D.L.; Stefanidis, D.; Lee, N.K. How resilient is your team? Exploring healthcare providers' well-being during the COVID-19 pandemic. *Am. J. Surg.* **2020**, *221*, 277–284.
8. Aggar, C.; Samios, C.; Penman, O.; Whiteing, N.; Massey, D.; Rafferty, R.; Bowen, K.; Stephens, A. The impact of COVID-19 pandemic-related stress experienced by Australian nurses. *Int. J. Ment. Health Nurs.* **2021**, *31*, 91–103.
9. Hammond, N.E.; Crowe, L.; Abbenbroek, B.; Elliott, R.; Tian, D.H.; Donaldson, L.H.; Fitzgerald, E.; Flower, O.; Grattan, S.; Harris, R.; et al. Impact of the coronavirus disease 2019 pandemic on critical care healthcare workers' depression, anxiety, and stress levels. *Aust. Crit. Care* **2020**, *34*, 146–154.
10. Holton, S.; Wynter, K.; Trueman, M.; Bruce, S.; Sweeney, S.; Crowe, S.; Dabscheck, A.; Eleftheriou, P.; Booth, S.; Hitch, D.; et al. Psychological well-being of Australian hospital clinical staff during the COVID-19 pandemic. *Aust. Health Rev.* **2020**, *45*, 297–305.

11. Axisa, C.; Nash, L.; Kelly, P.; Willcock, S. Burnout and distress in Australian physician trainees: Evaluation of a wellbeing workshop. *Australas. Psychiatry* **2019**, *27*, 255–261.
12. Wu, F.; Ireland, M.; Hafekost, K.; Lawrence, D. *National Mental Health Survey of Doctors and Medical Students*; Beyond Blue: Melbourne, Australia, 2013; p. 156.
13. Creedy, D.K.; Sidebotham, M.; Gamble, J.; Pallant, J.; Fenwick, J. Prevalence of burnout, depression, anxiety and stress in Australian midwives: A cross-sectional survey. *BMC Pregnancy Childbirth* **2017**, *17*, 13.
14. Hegney, D.G.; Craigie, M.; Hemsworth, D.; Osseiran-Moisson, R.; Aoun, S.; Francis, K.; Drury, V. Compassion satisfaction, compassion fatigue, anxiety, depression and stress in registered nurses in Australia: Study 1 results. *J. Nurs. Manag.* **2013**, *22*, 506–518.
15. Maharaj, S.; Lees, T.; Lal, S. Prevalence and Risk Factors of Depression, Anxiety, and Stress in a Cohort of Australian Nurses. *Int. J. Environ. Res. Public Health* **2019**, *16*, 61.
16. McGarry, S.; Girdler, S.; McDonald, A.; Valentine, J.; Lee, S.-L.; Blair, E.; Wood, F.; Elliott, C. Paediatric health-care professionals: Relationships between psychological distress, resilience and coping skills. *J. Paediatr. Child Health* **2013**, *49*, 725–732.
17. Pyper, Z.; Paterson, J.L. Fatigue and mental health in Australian rural and regional ambulance personnel. *Emerg. Med. Australas.* **2015**, *28*, 62–66.
18. Rossell, S.L.; Neill, E.; Phillipou, A.; Tan, E.J.; Toh, W.L.; E Van Rheenen, T.; Meyer, D. An overview of current mental health in the general population of Australia during the COVID-19 pandemic: Results from the COLLATE project. *Psychiatry Res.* **2020**, *296*, 113660.
19. Stanton, R.; To, Q.G.; Khalesi, S.; Williams, S.L.; Alley, S.J.; Thwaite, T.L.; Fenning, A.S.; Vandelanotte, C. Depression, Anxiety and stress during COVID-19: Associations with changes in physical activity, sleep, tobacco and alcohol use in australian adults. *Int. J. Environ. Res. Public Health* **2020**, *17*, 4065.
20. Sanghera, J.; Pattani, N.; Hashmi, Y.; Varley, K.F.; Cheruvu, M.S.; Bradley, A.; Burke, J.R. The impact of SARS-CoV-2 on the mental health of healthcare workers in a hospital setting—A Systematic Review. *J. Occup. Health* **2020**, *62*, e12175.
21. Serrano-Ripoll, M.J.; Meneses-Echavez, J.F.; Ricci-Cabello, I.; Fraile-Navarro, D.; Fiol-deRoque, M.A.; Pastor-Moreno, G.; Gonçalves-Bradley, D.C. Impact of viral epidemic outbreaks on mental health of healthcare workers: A rapid systematic review and meta-analysis. *J. Affect. Disord.* **2020**, *277*, 347–357.
22. Tran, T.D.; Hammarberg, K.; Kirkman, M.; Nguyen, H.T.M.; Fisher, J. Alcohol use and mental health status during the first months of COVID-19 pandemic in Australia. *J. Affect. Disord.* **2020**, *277*, 810–813.
23. Rahman, M.A.; Hoque, N.; Alif, S.M.; Salehin, M.; Islam, S.M.S.; Banik, B.; Sharif, A.; Nazim, N.B.; Sultana, F.; Cross, W. Factors associated with psychological distress, fear and coping strategies during the COVID-19 pandemic in Australia. *Glob. Health* **2020**, *16*, 95.
24. Bhoyroo, R.; Chivers, P.; Millar, L.; Bulsara, C.; Piggott, B.; Lambert, M.; Codde, J. Life in a time of COVID: A mixed method study of the changes in lifestyle, mental and psychosocial health during and after lockdown in Western Australians. *BMC Public Health* **2021**, *21*, 1947.
